# 

**Published:** 1894-08

**Authors:** 


### NOTICES.

### The American Dental Society of Europe.

Notice has already been given in the July number of the DENTAL REGISTER, of the nineteenth meeting of the American Dental Society of Europe, which is to be held in the *Athenee*, Geneva, Switzerland, August 6th, 7th and 8th inclusive.

The following list of papers to be read and discussed has been prepared, viz.: "A Few More Words on Pyorrhœa," by Dr. A. V. Elliott, of Florence; "Theory of the Crystal Mat

Gold," by Dr. De Trey, of Basle; "The Etiology of Defective Enamel," by Dr. Waldo E. Royce, of Tunbridge-Wells; "On the Treatment of Bicuspids," by Dr. Chas. W. Jenkins, of Zurich; "The Law of Statistics in Hard and Soft Dental Tissues Demonstrated by Anatomical and Microscopical Specimens, Drawings and Models," by Prof. August Eternod, of Geneva; "Copper Amalgam, Our Last and Most Unholy Transgression," by Dr. L. C. Bryan.

There will be a paper by Prof. W. D. Miller, of Berlin, and one by Prof. F. Vulliet.

CLINICS AND DEMONSTRATIONS.

"A Practical Method of Contouring Some Teeth with Gold," by Dr. H. L. Schaffner, of Florence; "Application of a New Set of Pluggers to the New Combined Sponge Mat Golds," by Dr. E. De Trey, of Basle; "The Basle Electric Engine Motor with Accumulators and Instantaneous Stop, Etc.," by Dr. Bryan, of Basle; "A Method of Fixing an Artificial Lower Incisor in Place of a Natural One Lost through Pyorrhœa," by Dr. Elliott, of Florence; "Demonstration of Cohesive and Non-Cohesive Gold Combined in Filling," by Dr. E. J. Wetzel., Mulhausen.

Dr. Luce, of Stuttgart, and Dr. Terry, of Milan, have kindly promised to furnish something of interest to the profession.

The Executive Committee have arranged for only two days of professional work, the other day to be spent in social recreation.

It will, without doubt, be a most enjoyable time for those who attend the meeting.

DR. LYMAN C. BRYAN, President, Basle.
DR. CHAS. W. JENKINS, Secretary, Zurich.

### Alumni Reunion.

There will be a reunion of the Alumni of the Dental Department of the University of Michigan at the time of the meeting of the American Dental Association, at Old Point Comfort, August 7th, 1894.

Come, let us renew old acquaintances and keep alive the interests of our Alma Mater.

U. D. BILLMEYER, Sec'y, Chattanooga, Tenn.
M. F. FINLEY, Pres't, Washington, D. C.

### Connecticut State Dental Association.

The Connecticut State Dental Association holds its next regular meeting in Hartford, the Third Tuesday in May, 1895. There will probably be a special meeeting December 11th, to celebrate the Semi-Centennial of the discovery of anæsthesia, by Horace Wells, a Hartford dentist.

The following officers were elected for the ensuing year: President, Dr. Charles P. Graham, Middletown; Vice-President, C. W. Strang, D.D.S., Bridgeport; Secretary, George L. Parmele, M.D.,D.M.D. Hartford; Treasurer, Daniel A. Jones, M.D. D.M.D., New Haven; Ass't Secretary, Charles McManus, D.D.S. Hartford. Executive Committee: Charles McManus, D.D.S., Chairman; Wm. H. Rider, M.D.S., Danbury; Dr. C. C. Barker Meriden.

G. L. PARMELE Secretary.

### THE DENTAL REGISTER,

**A MONTHLY MAGAZINE DEVOTED TO THE INTERESTS OF THE**

### DENTAL PROFESSION.

*Terms Reduced to* $2.00 *Per Year. Single Copies*, 25 *Cents.*

**EDITED BY PROF. J. TAFT, D.D.S.**

—AND—

**Published by SAM'L A. CROCKER & CO., Ohio Dental and Surgical Depot.**

The volume commences in January and closes in December.

The REGISTER being issued monthly is always fresh, therefore Indispensable to the practitioner who desires to keep posted up with the new inventions and improvements which are daily developed. Neither expense nor effort will be spared to give value and variety to its contents. Articles will be illustrated with well executed engravings whenever they will add to the interest or assist to explanation.

Its extensive circulation and frequency of issue make it one of the BEST DENTAL ADVERTISING MEDIUMS in the United States.

All subscriptions must begin with January or July.

Local or special notices, five lines or less, will be inserted for $3 each insertion; over five lines, 50 cts. per line.

All advertising bills payable in advance, or at furthest, after the issue of the first number containing the advertisement. All transient advertisements to be paid for in advance.

☞CONTRIBUTIONS SOLICITED.

Exclusively Editorial Communications should be addressed to the Editor.

The latest number of the REGISTER may be ordered with or without other goods at any time, and all letters should be addressed to

